# The PhoBR two-component system regulates antibiotic biosynthesis in *Serratia *in response to phosphate

**DOI:** 10.1186/1471-2180-9-112

**Published:** 2009-05-28

**Authors:** Tamzin Gristwood, Peter C Fineran, Lee Everson, Neil R Williamson, George P Salmond

**Affiliations:** 1 Department of Biochemistry, University of Cambridge, Cambridge, CB2 1QW, UK; 2 Department of Microbiology & Immunology, University of Otago, PO Box 56, Dunedin, New Zealand

## Abstract

**Background:**

Secondary metabolism in *Serratia *sp. ATCC 39006 (*Serratia *39006) is controlled via a complex network of regulators, including a LuxIR-type (SmaIR) quorum sensing (QS) system. Here we investigate the molecular mechanism by which phosphate limitation controls biosynthesis of two antibiotic secondary metabolites, prodigiosin and carbapenem, in *Serratia *39006.

**Results:**

We demonstrate that a mutation in the high affinity phosphate transporter *pstSCAB-phoU*, believed to mimic low phosphate conditions, causes upregulation of secondary metabolism and QS in *Serratia *39006, via the PhoBR two-component system. Phosphate limitation also activated secondary metabolism and QS in *Serratia *39006. In addition, a *pstS *mutation resulted in upregulation of *rap*. Rap, a putative SlyA/MarR-family transcriptional regulator, shares similarity with the global regulator RovA (regulator of virulence) from *Yersina *spp. and is an activator of secondary metabolism in *Serratia *39006. We demonstrate that expression of *rap*, *pigA-O *(encoding the prodigiosin biosynthetic operon) and *smaI *are controlled via PhoBR in *Serratia *39006.

**Conclusion:**

Phosphate limitation regulates secondary metabolism in *Serratia *39006 via multiple inter-linked pathways, incorporating transcriptional control mediated by three important global regulators, PhoB, SmaR and Rap.

## Background

Phosphate is an essential component of numerous biomolecules. Therefore, the control of intracellular phosphate concentrations is vital for bacterial survival. At least two major systems are involved in managing intracellular concentrations of inorganic orthophosphate (P_i_), the preferred primary source of phosphate [1]. When P_i _is abundant, the low affinity Pit transporter appears to be primarily responsible for P_i _uptake [2-4]. When P_i _becomes limited, the high affinity Pst transport system (PstSCAB-PhoU) is activated, and takes over as the predominant P_i _uptake system [5-8].

In *Escherichia coli *and other Enterobacteriaceae, the cellular response to P_i _availability is mediated via the PhoBR two-component system. Under conditions of P_i _limitation, the sensor histidine kinase PhoR is autophosphorylated [9]. PhoR then activates its cognate response regulator, PhoB [9], which in turn activates expression of multiple genes, termed the Pho regulon, via direct binding to a conserved Pho box sequence found overlapping -35 regions in target gene promoters [10-12]. In *E. coli*, the Pho regulon is believed to consist of approximately 30 genes involved in the adaptation to survival under low P_i _conditions, including *pstSCAB-phoU *and *phoBR *[1]. Phosphate regulation is controlled via similar mechanisms in *Bacillus subtilis *and *Streptomyces *species, although the consensus Pho boxes are different in each system [13,14]. Mutations in the *pstSCAB-phoU *operon result in constitutive activation of PhoR and hence, constitutive phosphorylation of PhoB [15,16]. Therefore, *pst *mutants are proposed to mimic low P_i _conditions.

P_i _has been found to negatively regulate the biosynthesis of antibiotics and other secondary metabolites in multiple bacterial species (reviewed in [17]). However, the complex molecular mechanisms underlying the P_i _mediated regulation of secondary metabolism are not well characterised. In this study we investigate the role of the PhoBR two-component system, and P_i _availability, on the regulation of antibiotic production in the Gram-negative Enterobacteriaceae, *Serratia *sp. ATCC 39006 (*Serratia *39006). *Serratia *39006 synthesises the red, tripyrrole antibiotic, prodigiosin (Pig; 2-methyl-3-pentyl-6-methoxyprodigiosin) [18]. The natural physiological role of Pig in the producing organism may be as an antimicrobial agent [19]. In addition, Pig is of clinical interest due to the observed anticancer and immunosuppressive properties of this compound [20-22]. *Serratia *39006 also produces the β-lactam antibiotic, carbapenem (Car; 1-carbapen-2-em-3-carboxylic acid) [23,24]. Both the Pig and Car biosynthetic gene clusters have been characterised (*pigA-O *and *carA-H*, respectively) [25,26].

Production of secondary metabolites in *Serratia *39006 is controlled by a hierarchial network of regulators [27]. This includes a LuxIR-type quorum sensing (QS) system (SmaIR) [25,28,29], which allows gene expression to be regulated in response to cell density via the production and detection of low molecular weight signal molecules [30]. In *Serratia *39006, the *N*-acyl homoserine lactone (AHL) synthase SmaI produces two signalling molecules, *N*-butanoyl-L-homoserine lactone (BHL) and *N*-hexanoyl-L-homoserine lactone (HHL), with BHL being the major product [25]. At low cell density, SmaR acts as a transcriptional repressor of target genes [28,29]. At high cell density, and hence high BHL/HHL levels, SmaR binds BHL/HHL, resulting in decreased DNA-binding affinity with a consequent alleviation of repression. QS controls secondary metabolism in *Serratia *39006 via at least four other regulatory genes (*carR*, *pigQ*, *pigR *and *rap*) [28,29]. The putative SlyA/MarR-family transcriptional regulator, Rap (regulator of antibiotic and pigment), is an activator of Pig and Car production in *Serratia *39006 [31]. Rap shares similarity with the global transcriptional regulator RovA (regulator of virulence) from *Yersina *spp. [32-34]. More than 20 additional genes have been shown to regulate secondary metabolism in *Serratia *39006, and these are predicted to be responding to additional environmental stimuli [19,27,35,36].

Previously, we demonstrated that, in *Serratia *39006, mutations within genes predicted to encode homologues of the *E. coli *PstSCAB-PhoU phosphate transport system resulted in over-production of both Pig (10-fold) and Car (2-fold), at the level of transcription of the biosynthetic genes [29]. In this study we investigate further the molecular mechanism by which these effects are occurring. We demonstrate that secondary metabolism in *Serratia *39006 is upregulated in response to mutations in PstSCAB-PhoU or P_i _limitation, via the PhoBR two-component system. In addition, we provide evidence that expression of the *smaI, pigA *and *rap *genes are activated via PhoBR in *Serratia *39006. Hence, we propose a model in which P_i _limitation increases secondary metabolism in *Serratia *39006 via multiple, inter-linked pathways, incorporating the global transcriptional regulators PhoB, SmaR and Rap.

## Results

### Sequence analysis of the *pstSCAB-phoU *operon in *Serratia *39006

**Figure 1:**
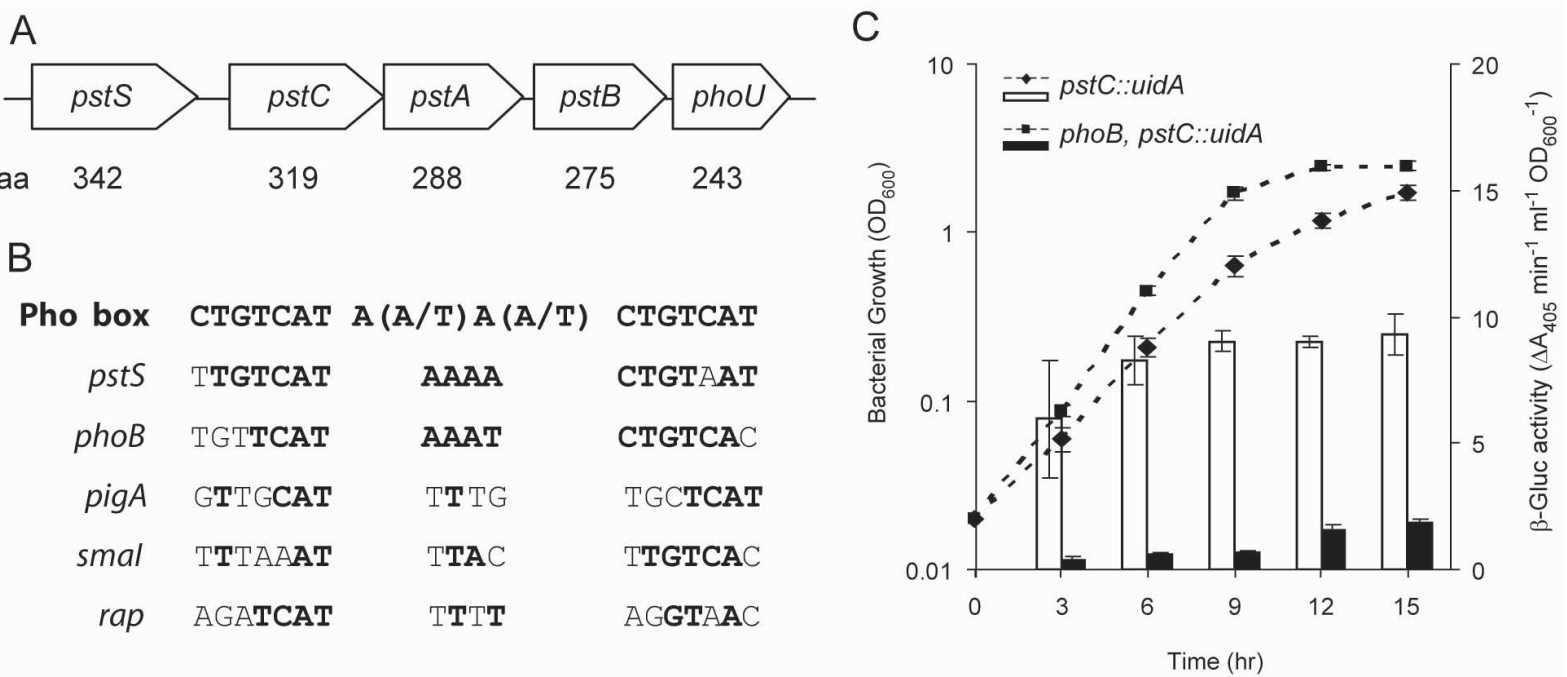
**The *Serratia *39006 Pst transporter is regulated via PhoBR.**. A) The *Serratia *39006 *pstSCAB-phoU *genes. (B) Putative Pho boxes found upstream of the *pstS*, *phoB*, *pigA*, *smaI *and *rap *genes in *Serratia *39006. The *E. coli *Pho box consensus sequence is shown [10-12]. Conserved nucleotides are shown in bold. (C) β-Glucuronidase activity was assayed throughout growth in LB from a chromosomal *pstC::uidA *fusion in an otherwise WT background (NW201; diamonds and open bars) or a *phoB *mutant background (NW202; squares and solid bars). Bars represent β-glucuronidase assays and dashed lines represent bacterial growth.

Previously, *Serratia *39006 mutants were identified which contained transposon insertions in regions sharing sequence similarity to the *pstS *and *pstA *genes from *E. coli *[29]. DNA sequencing analysis of this region revealed that *Serratia *39006 possesses a complete *pstSCAB-phoU *operon, the organisation of which is consistent with other enteric bacteria in which a *pst *operon has been identified (Fig. 1A).

The *Serratia *39006 *pstS *gene was predicted to encode a protein most similar to PstS from the enteric bacteria *Erwinia carotovora *ssp. *atroseptica *SCRI1043 (*Eca *1043) (82% identity/90% similarity). The putative protein product encoded by *pstC *shared 90% identity and 95% similarity with PstC of *Eca *1043. The *pstA *gene is predicted to encode a protein most similar to PstA of *Eca *1043 (87% identity/92% similarity). The predicted protein encoded by *pstB *was most similar to PstB of *Eca *1043 (88% identity/91% similarity). Finally, *phoU *was predicted to encode a protein most similar to PhoU of *Eca *1043 (94% identity/98% similarity).

### Isolation and sequence analysis of *phoBR *mutants of *Serratia *39006

Mutations in the *pstSCAB-phoU *operon are thought to mimic growth in limiting phosphate, and hence result in constitutive activation of the Pho regulon [15]. We previously showed that Pig, Car and AHL production were increased in the *pstS *mutant [29]. A possible explanation for this effect is that *pigA, carA *and *smaI *are regulated via the *Serratia *39006 Pho regulon.

Random transposon insertions in the *phoBR *operon were isolated based on their lack of hyperpigmentation when grown on P_i_-limiting media. Growth on P_i_-limiting media results in increased Pig production in the wild-type (WT; throughout this manuscript WT refers to the LacA parental strain) [29]. Potential *phoBR *mutants were then checked for their loss of alkaline phosphatase activity (*phoA*, encoding alkaline phosphatase, is a conserved Pho regulon gene [1,37]) and the sequence of the operon was determined, as described in *Methods*. The *phoB *gene was predicted to encode a 229 amino acid (aa) protein with highest similarity to PhoB from *Eca *1043 (96% identity/98% similarity). The *phoR *gene was located 28 bp downstream of *phoB*, and was predicted to encode a 440 aa protein sharing the highest degree of similarity to *Eca *1043 PhoR (87% identity/90% similarity).

### PhoB regulates expression of *pstC *in *Serratia *39006

In *E. coli*, the *pst *operon is activated via direct binding of PhoB to a conserved Pho box upstream of *pstS *[10-12]. As *Serratia *39006 is a member of the Enterobacteriaceae, we identified potential Pho boxes based on the *E. coli *consensus sequence. A potential Pho box was identified within the *pstS *promoter region of *Serratia *39006, centred 122 bp upstream of the *pstS *start codon (Fig. 1B). This suggested that, as could be expected based on regulation of the *pstSCAB-phoU *genes in other bacteria, the *pstSCAB-phoU *genes in *Serratia *39006 may be regulated by PhoB. A putative Pho box was also identified upstream of *phoB *(Fig 1B), centred 68 bp upstream of the *phoB *start codon, suggesting that *phoBR *may be auto-regulated via the putative Pho box.

β-Glucuronidase activity produced from a chromosomal *pstC::uidA *transcriptional fusion was measured in the presence or absence of a secondary mutation in *phoB*. The *pstC::uidA *fusion strain does not contain a functional Pst transporter and is therefore believed to mimic low phosphate conditions. These data showed that, in the presence of functional PhoB, *pstC *was expressed constitutively throughout growth (Fig. 1C). Expression was dramatically reduced following inactivation of *phoB*, indicating that PhoB activates expression of the *pst *operon in *Serratia *39006 (Fig. 1C).

### Insertions within *phoBR *abolish upregulation of secondary metabolism and QS in the *pstS *mutant

**Figure 2:**
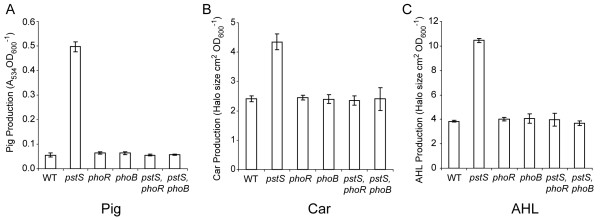
**The effects of a *pstS *mutation on secondary metabolism and QS are occurring via PhoBR**. (A) Pig, (B) Car and (C) AHL production were measured from WT, *pstS *mutant (ROP2), *phoR *mutant (BR1), *phoB *mutant (BR9), *pstS, phoR *double mutant (PCF60) and *pstS*, *phoB *double mutant (PCF59) cells. Production was assayed from cells grown to early stationary phase in LB.

It was hypothesised that the upregulation of Pig, Car and QS in a *Serratia *39006 *pst *mutant was mediated via the PhoBR two-component system. Assessment of Pig, Car and QS phenotypes in *pstS*, *phoB *and *pstS*, *phoR *double mutants confirmed that *phoB *and *phoR *were responsible for the upregulation of secondary metabolism in a *pstS *mutant background. The *pstS *mutant was increased for Pig (9-fold), Car (2-fold) and AHL (2.5-fold) production compared with the WT (Fig. 2). However, the *pstS*, *phoB *and *pstS*, *phoR *double mutants were restored to WT levels for Pig, Car and AHL production in LB (Fig. 2). Single *phoB *or *phoR *mutations had no effect on Pig, Car or AHL production (Fig. 2). As it has been previously shown that upregulation of Car in response to a *pst *mutation is mediated via the upregulation of QS [29], we focused on the effects on *pigA *and *smaI *expression for the remainder of this study.

### Insertions within *phoBR *abolish transcriptional upregulation of *pigA *and *smaI *in the *pstS *mutant

**Figure 3:**
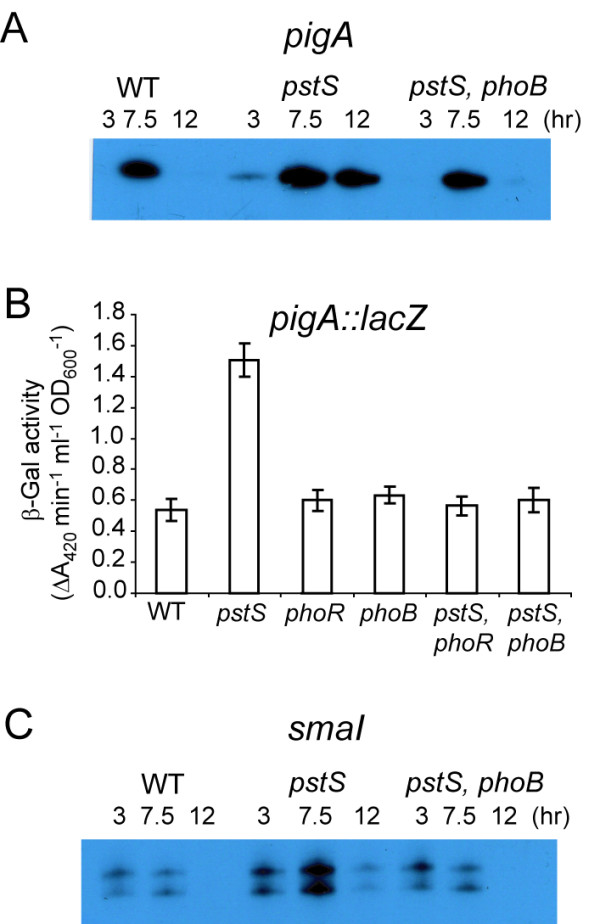
**A *pstS *mutation effects transcription of *pigA *and *smaI *via PhoBR**. Primer extension analysis was used to measure the level of (A) *pigA *or (C) *smaI *transcripts in WT, *pstS *mutant (ROP2), or *pstS, phoB *(RBR9) double mutant strains throughout growth in LB. (B) β-Galactosidase activity was measured from a chromosomal *pigA::lacZ *fusion in an otherwise WT background (NW60), or in *pstS *(PCF76), *phoR *(PCF75), *phoB *(PCF74), *pstS*, *phoR *double (PCF78) or *pstS*, *phoB *double (PCF77) mutant backgrounds. Activity was assayed from cells grown to early stationary phase in LB.

Phenotypic analysis showed that PhoBR are required for the upregulation of secondary metabolism and QS in response to mutation of the *pstSCAB-phoU *operon (described above). To confirm that these effects are exerted at the transcriptional level, primer extension analysis was used to assess levels of the *pigA *and *smaI *transcripts throughout growth in WT, *pstS *mutant and *pstS*, *phoB *double mutant strains. The abundance of *pigA *mRNA in the *pstS*, *phoB *double mutant was restored to levels similar to those displayed in WT *Serratia *39006 (Fig. 3A). A chromosomal *pigA::lacZ *transcriptional fusion was used to confirm this result; a 3-fold increase in *pigA *transcription was observed in a *pstS *mutant, this was restored to WT levels following a secondary mutation in *phoB *or *phoR *(Fig. 3B). The upregulation of *smaI *transcription in the *pstS *mutant was also abolished by a *phoB *mutation (Fig. 3C). This is consistent with the hypothesis that PhoB, either directly or indirectly, activates expression of *pigA *and *smaI *in response to constitutive phosphorylation by PhoR as a result of the *pstS *insertion.

### Insertions within *pstSCAB-phoU *result in increased transcription of *rap*

**Figure 4:**
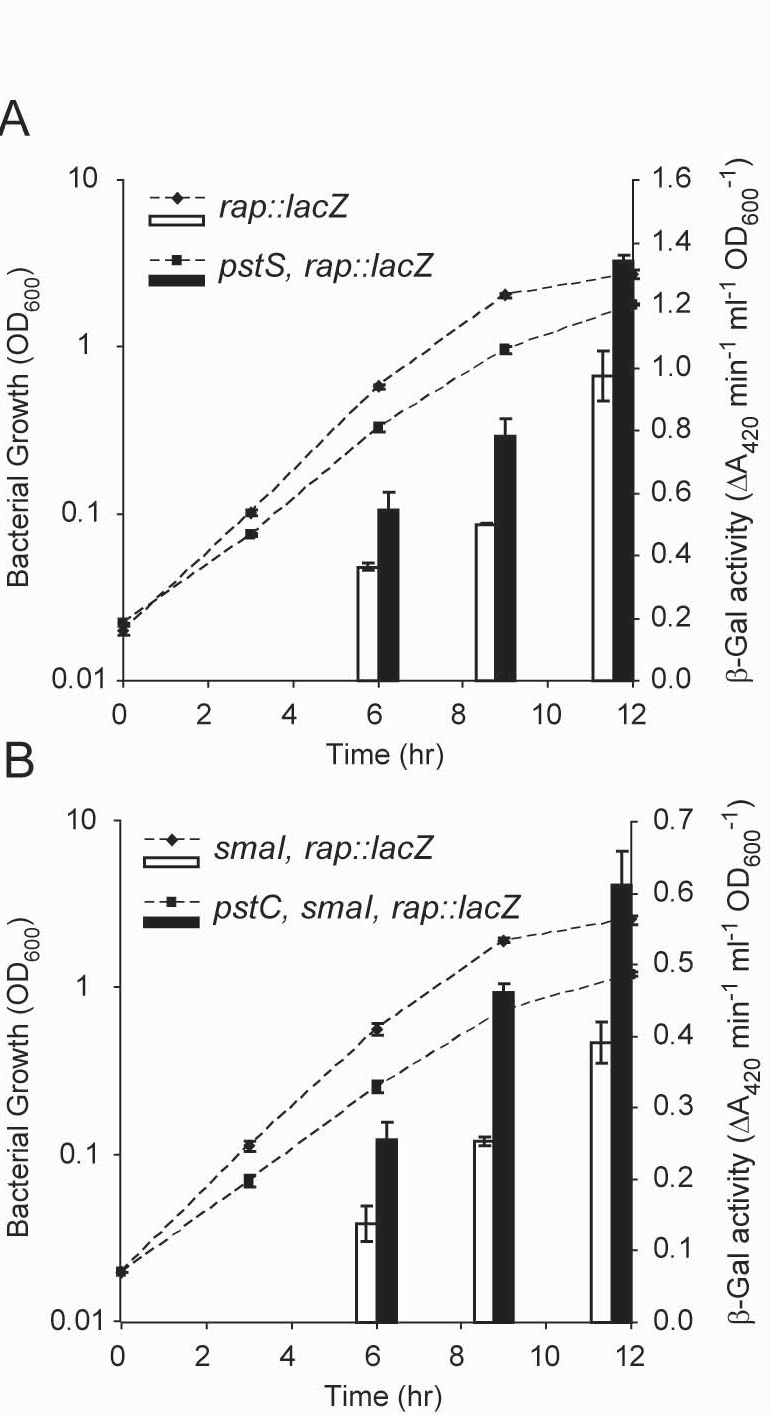
**Expression of *rap *is activated following mutation of the *pstSCAB *operon**. β-Galactosidase activity was assayed throughout growth from a chromosomal *rap::lacZ *fusion in (A) an otherwise WT background (RAPL;diamonds and open bars) or a *pstS *mutant background (PCF45; squares and solid bars), or (B) a *smaI *(ISRL;diamonds and open bars) or *pstC*, *smaI *(TG71; squares and solid bars) mutant background. In both graphs, bars represent β-galactosidase assays and dashed lines represent bacterial growth.

A complex network of regulators controls secondary metabolism in *Serratia *39006 [27]. Therefore, it was possible that the effects on Pig and AHL production, in response to a *pst *mutation, were mediated via one or more of these regulators. To test if the effect on *smaI *and *pigA *transcription was mediated through any of the known secondary metabolite regulators, the expression of chromosomal *lacZ *transcriptional fusions in *pigP*, *pigQ*, *pigR*, *pigS*, *pigT*, *pigV*, *pigW*, *pigX*, *pigZ*, *rap *and *carR *was assessed throughout growth in the presence or absence of a *pstS*::mini-Tn*5*Sm/Sp insertion (data not shown). No effect was seen on any of the regulatory genes except for *rap*. The expression of *rap *was increased by 1.4-fold in the *pstS *mutant (Fig. 4A). Rap is a putative SlyA/MarR-family transcriptional regulator. As expression of *rap *is known to be regulated by QS [28], the effect of a *pstC *mutation on expression of a *rap::lacZ *transcriptional fusion was assessed in a *smaI *mutant background. A mutation within the *pstSCAB-phoU *operon was still able to activate *rap *transcription (1.5-fold increase), in the absence of functional *smaI*, indicating that this effect is via both QS -dependent and -independent pathways (Fig. 4B).

### PhoB activates expression from the *pigA *and *rap *promoters in an *E. coli *system

**Figure 5:**
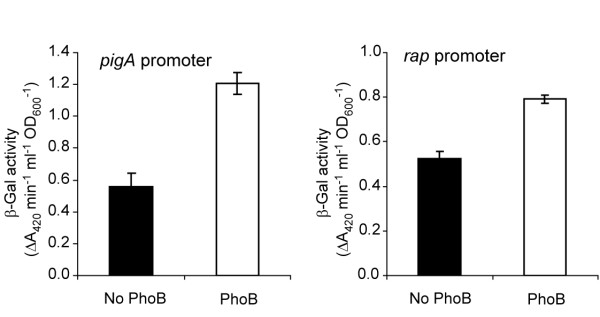
**In *E. coli*, *Serratia *39006 PhoB can activate expression from the *pigA *and *rap *promoters**. β-Galactosidase activity was measured from *E. coli *cells grown in LB carrying plasmid pTA15 or pTA14 (containing the *pigA *or *rap *promoters respectively cloned upstream of a promoterless *lacZ *gene) and either an empty vector control (pQE-80L) (solid bar) or pTA74, encoding PhoB (unfilled bar).

To investigate the control of the *pigA*, *rap *and *smaI *promoters in more detail, an *E. coli *plasmid-based system was used (described in *Methods*). β-Galactosidase activity was measured from *E. coli *strains carrying the *pigA*, *rap *or *smaI *promoters, inserted upstream of a promoterless *lacZ *gene (encoded by vectors pTA15, pTA14 or pTG27, respectively) in the presence or absence of *Serratia *39006 PhoB, encoded by plasmid pTA74. Transcription from the *pigA *and *rap *promoters increased in the presence of pTA74, indicating that these genes may be activated by PhoB (Fig. 5). Unfortunately, the level of expression from the *smaI *promoter was negligible in this system (data not shown). Therefore, it was not possible to determine whether PhoB was modulating transcription from the *smaI *promoter. In the *E. coli *system, the degree of activation from both the *pigA *and *rap *promoters in the presence of PhoB is comparable with the levels of activation observed using chromosomal *pigA::lacZ *and *rap::lacZ *transcriptional fusions as a result of *pstS*/*pstC *mutation in *Serratia *39006 (Fig. 3B & Fig. 4). Putative weak Pho boxes were identified within the promoter regions of *pigA *and *smaI*, overlapping the predicted -35 sequences and centred 28 bp and 34 bp, respectively, upstream of the transcriptional start sites, which were previously mapped by primer extension [29] (Fig. 1B). A putative weak Pho box was also identified within the *rap *promoter, centred 148 bp upstream of the *rap *start codon (Fig. 1B). The presence of putative Pho boxes suggest that PhoB may directly activate expression of *pigA*, *smaI *and *rap*, although this has not yet been shown experimentally. In the *E. coli *reporter assays described, it is possible that *Serratia *39006 PhoB may show activity in the absence of the cognate *Serratia *39006 histidine kinase, PhoR, due to cross-regulation by non-cognate *E. coli *histidine kinases, or as a result of low level activity of the unphosphorylated *Serratia *39006 PhoB.

### P_i _regulates secondary metabolism and QS in *Serratia *39006

**Figure 6:**
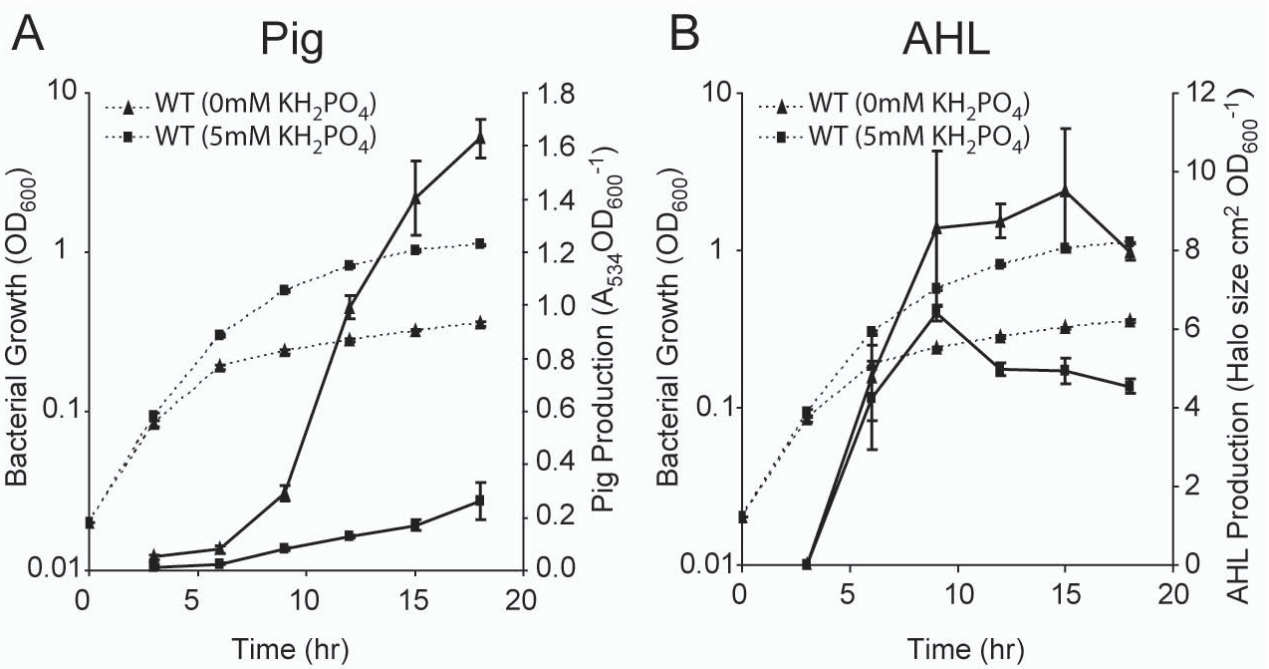
**P_i _limitation affects secondary metabolism and QS**. (A) Pig and (B) AHL production in WT cells were measured throughout growth in phosphate-limiting medium with (squares) or without (triangles) the addition of 5 mM KH_2_PO_4_. In all graphs, solid lines represent Pig or AHL assays and dashed lines represent bacterial growth.

**Figure 7:**
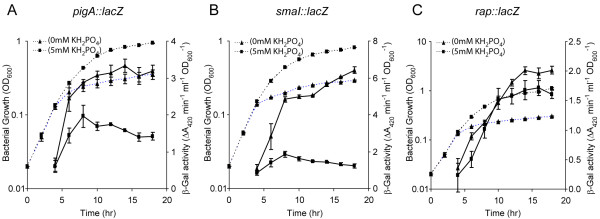
**The effect of P_i _limitation on *pigA*, *smaI *and *rap *transcription**. β-Galactosidase activity was measured from a chromosomal (A) *pigA::lacZ *(MCP2L), (B) *smaI::lacZ *(LC13) or (C) *rap::lacZ *(RAPL) strain throughout growth in phosphate-limiting medium with (squares) or without (triangles) the addition of 5 mM KH_2_PO_4_. In all graphs, solid lines represent β-galactosidase assays and dashed lines represent bacterial growth.

In other species, PhoBR upregulates expression of multiple genes when the cell is starved for P_i _. As P_i _has been shown to control secondary metabolism in multiple species [17], we investigated whether secondary metabolism and QS in *Serratia *39006 were also modified by P_i _limitation. Growth of *Serratia *39006 in phosphate-limiting medium (PL medium) without the addition of 5 mM KH_2_PO_4 _resulted in an increase in Pig (6-fold) and AHL (2-fold) production (Fig. 6A &6B), reminiscent of the effects of *pstS *mutations. β-Galactosidase activity from strains containing chromosomal *pigA*::*lacZ*, *smaI::lacZ *and *rap::lacZ *fusions grown in PL medium without the addition of 5 mM KH_2_PO_4 _was also assessed. P_i _limitation resulted in increased transcription of *pigA *(2-fold) and *smaI *(5-fold) compared with P_i _replete conditions (Fig. 7A &7B), although there was not a clear increase in *rap *transcription (Fig. 7C). These experiments demonstrate that low P_i_, like *pstSCAB-phoU *mutations, controls the transcription of *pigA *and *smaI *to up-regulate secondary metabolism and QS. However, in each instance, the fold increase in response to P_i _limitation is lower (by approximately 35%) than that observed in a *pst *mutant. As the increase in *rap *transcription in a *pst *mutant is below 2-fold, a lesser change, in response to P_i _limitation, may be below the level of detection.

**Figure 8:**
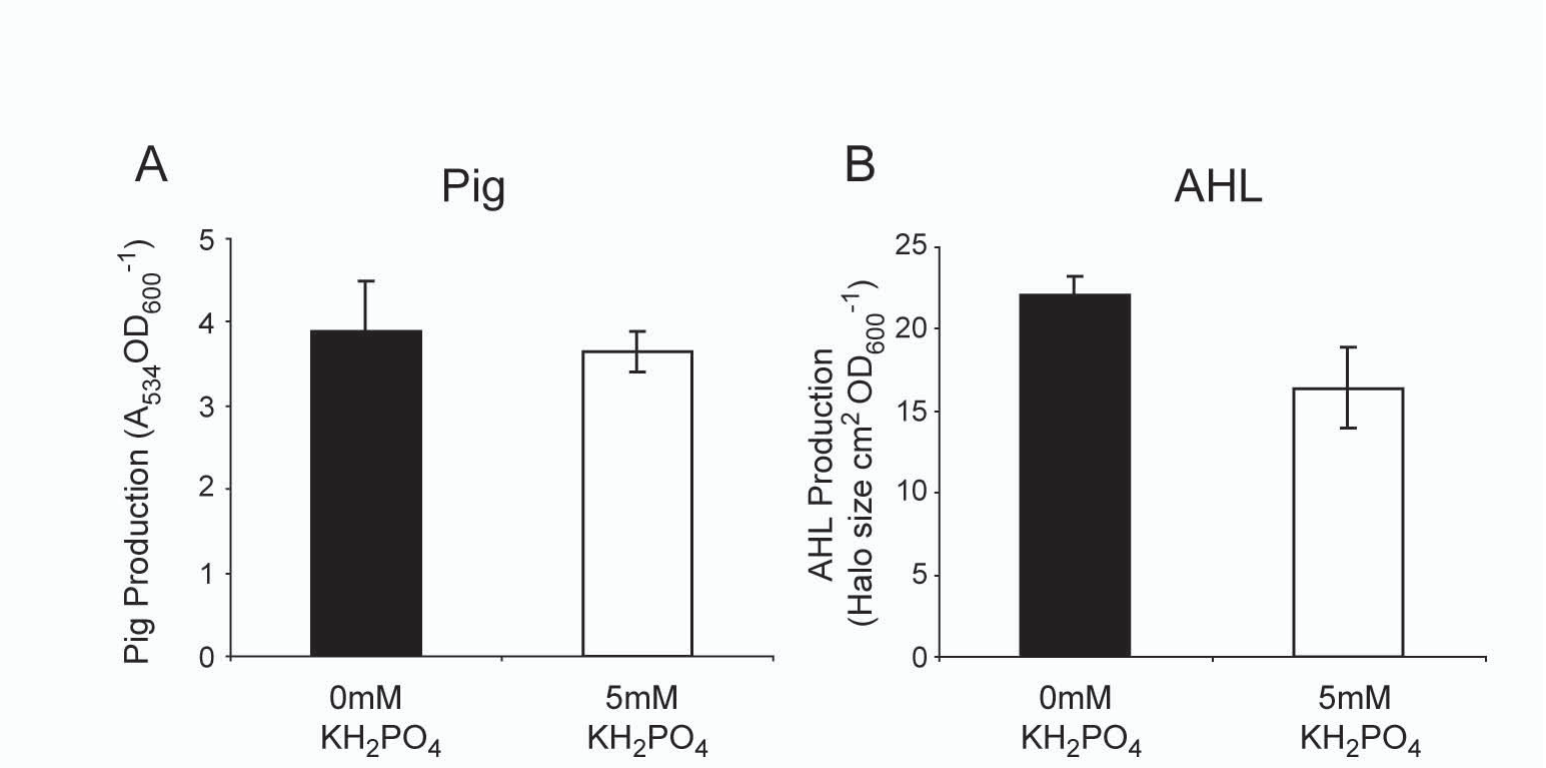
**A *pstS *mutant is largely unresponsive to P_i _limitation**. (A) Pig and (B) AHL production was measured from a *pstS *mutant (ROP2) grown to early stationary phase in phosphate-limiting medium with (open bars) or without (solid bars) the addition of 5 mM KH_2_PO_4_.

We predicted that a *pstS *mutation would be epistatic to the effects of P_i _on secondary metabolism and QS. In a *pstS *mutant, P_i _limitation did not result in an increase in maximal Pig production (Fig. 8A), although slightly premature production of Pig was observed (data not shown). In addition, P_i _limitation resulted in only a small (1.3-fold) increase in AHL production in a *pstS *mutant (Fig. 8B). Taken together, the data suggest that in *Serratia *39006, as in other bacteria, mutation of *pstS *mimics the effect of P_i_-limiting media. However, other mechanisms also appear to play a role, facilitating the small increase in AHL production observed in response to P_i _limitation despite the absence of a functional PstSCAB-PhoU system.

## Discussion

There are multiple studies identifying environmental factors that effect Pig production in *Serratia *spp., including the effects of salt concentration, temperature, oxygen availability and multiple metal ion concentrations [27]. However, the molecular mechanism underlying most of these responses has not been elucidated. Here, we investigate the molecular mechanism by which P_i _limitation affects secondary metabolism in the enteric bacteria *Serratia *39006.

It was previously shown that a *pstS *mutation in *Serratia *39006 resulted in the upregulation of QS and secondary metabolism [29]. Here, we demonstrate that these effects are occurring via the PhoBR two-component system, since a secondary mutation in *phoBR *abolished the effects of a *pstS *mutation. In addition, we confirm that QS and secondary metabolism are upregulated in response to P_i _limitation, and that this is occurring primarily via the PstSCAB-PhoU transport system. We also demonstrate that expression of *rap *is upregulated in response to a *pstS *mutation. Rap is an activator of Pig and Car, and a repressor of surfactant production and swarming motility, in *Serratia *39006 [19,29]. Rap shares similarity with the SlyA/MarR-family global transcription factor, RovA, which regulates genes required for host colonization in *Yersinia *spp. [32-34]. Therefore, our results indicate that three global transcriptional regulators, Rap, SmaR and PhoB, are involved in mediating the effects of P_i _limitation on secondary metabolism in *Serratia *39006.

A mutation of the *pstSCAB-phoU *genes resulted in a clear increase in Pig and AHL production, and a clear increase in *pigA*, *smaI *and *rap *transcription. However, following P_i _limitation, the effects on secondary metabolism and gene expression were less dramatic. The degree of activation of Pig and AHL production, and *pigA *transcription, was approximately 35% lower following P_i _limitation than the levels of activation observed in a *pstS *mutant. In addition, a clear increase in *rap *transcription was not observed following P_i _limitation. It is possible that this reduced effect is due to the fact that a *pstS *mutant is constitutively mimicking extreme P_i _limitation. However, when WT cells are transferred to phosphate limiting media, there may be phosphate carry over from the initial inoculum, and the cells may utilise existing intracellular phosphate stores, for example inorganic polyphosphate, before phosphate starvation occurs. As the increase in *rap *transcription in a *pstS *mutant is below 2-fold, we believe that a 35% reduction in activation, in response to P_i _limitation, may be undetectable. An alternative explanation could be that *rap *is induced via PhoBR, but not in response to P_i _limitation. Previously, PhoBR has been shown to activate expression of the *asr *(acid shock RNA) gene, but P_i _limitation did not activate *asr *expression [38]. In addition, there is also evidence that PhoB can be activated by non-partner histidine kinases, in the absence of PhoR [39]. This has lead to the hypothesis that PhoBR may activate genes in response to a variety of environmental cues, in addition to P_i _limitation [39].

It may not be entirely accurate to describe the effect of a *pstS *mutation, or P_i _limitation, on QS as 'upregulation'. For QS to function correctly, it is the absolute concentrations of the AHL signal molecule that is critical, not the amount per cell [30]. Due to the growth defect observed following a *pstS *mutation or P_i _limitation, the amount of AHL per cell is increased, but the absolute value remains comparable to WT/P_i _excess conditions. Therefore, it may be more accurate to state that the upregulation of *smaI *transcription, following *pstS *mutation or P_i _limitation, allows maintenance of QS regulon control despite the reduced growth rate. This idea is supported by the fact that although *carR*, *pigQ*, *pigR *and *rap *are all regulated by QS in *Serratia *39006 [28,29], only *rap *transcription is upregulated in response to a *pstS *mutation. Our experiments indicate that, in response to a *pst *mutation, *rap *is activated independently of QS, and that activation may be mediated via PhoB.

Activation of *carA *expression, following *pstS *mutation, was previously reported to be dependent on the upregulation of QS [29]. However, as Rap is also an activator of *carA *transcription [29], it is possible that Rap, rather than QS, is responsible for the activation of *carA *following a *pstS *mutation. We propose that a dual mechanism, involving (1) the alleviation of SmaR repression at lower cell density, via upregulation of *smaI*, and (2) increased levels of Rap via PhoB mediated transcriptional activation, is responsible for the increase in *carA *expression following *pstS *mutation. In the absence of AHL (and hence constitutive SmaR repression), *carA *transcription is essentially abolished [29] and hence, further activation by Rap, in response to a *pstS *mutation, might not be possible.

**Figure 9:**
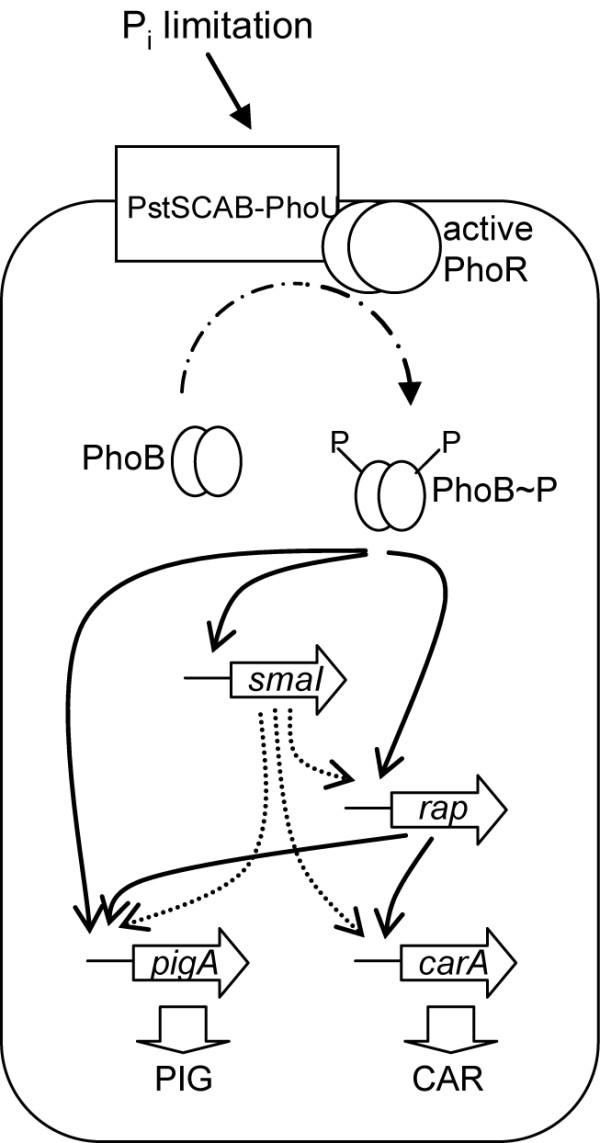
**The proposed mechanism by P_i _limitation can upregulate secondary metabolism in *Serratia *39006**. In response to P_i _limitation (or *pstS *mutation), PhoR activates PhoB by phosphorylation. Active PhoB can then activate transcription of *smaI*, *pigA *and *rap *(indicated using solid arrows). Upregulation of *smaI *results in activation of the QS regulated genes (*pigA*, *carA *and *rap*), via AHL mediated SmaR derepression (indicated using dashed arrows). Rap then further activates *carA *and *pigA *expression (indicated using solid arrows). This results in upregulation of Pig and Car production.

Based on our data, we propose a model by which P_i _limitation results in upregulation of secondary metabolism via multiple inter-linked pathways (Fig. 9). In response to P_i _limitation, or following mutation of the *pstSCAB *genes, PhoB is activated by phosphorylation [9,15,16]. PhoB~P can then activate expression of genes involved in the *Serratia *phosphate response which includes *smaI*, *pigA *and *rap*. Activation of *pigA *expression causes increased Pig production. Upregulation of *smaI *allows appropriate derepression by SmaR [28,29]. This allows activation of *pigA*, *carA *and *rap *transcription. Rap, which is activated via QS and the phosphate response, can then further activate *carA *and *pigA *transcription. This results in upregulation of both Car and Pig production via multiple pathways.

Multiple studies have linked P_i _limitation to enhanced secondary metabolite production [17]. However, the complex molecular mechanisms underlying phosphate-mediated regulation have proven difficult to elucidate. Extensive studies in *Streptomyces *species have shown that PhoPR (PhoBR) activates secondary metabolism in response to P_i _limitation, including biosynthesis of undecylprodigiosin, a tripyrrole closely related to Pig [40,41]. However, in *Streptomyces*, inactivation of PhoP or deletion of *phoPR *also activates secondary metabolism [41]. In contrast, deletion of *phoB *and/or *phoR *in *Serratia *39006 had no impact on secondary metabolism, demonstrating clear differences between the regulatory mechanisms employed by these distantly related bacteria. Although the requirement for increased secondary metabolism under conditions of phosphate limitation is unclear, it has been proposed that enhanced secondary metabolism allows the production of compounds which may, for example, directly antagonise other microorganisms or act as signalling molecules, thereby providing producing organisms with a competitive advantage under nutrient deprived conditions [40,42,43].

## Conclusion

In conclusion, we have established that via the global transcriptional regulators PhoB, SmaR and Rap, multiple inter-linked pathways are acting to upregulate secondary metabolism in *Serratia *39006 under conditions of P_i _limitation, highlighting the importance of Pig and Car production under these conditions.

## Methods

### Bacterial strains, plasmids, phage and culture conditions

Bacterial strains and plasmids are listed in Additional File 1[44-49]. *Serratia *sp. ATCC 39006 derivative strains were grown at 30°C and *E. coli *strains were grown at 37°C in Luria broth (LB; 5 g l^-1 ^yeast extract, 10 g l^-1 ^bacto tryptone and 5 g l^-1 ^NaCl), minimal media (0.1% w/v (NH_4_)_2_SO_4_, 0.41 mM MgSO_4_, 0.2% w/v glucose, 40 mM K_2_HPO_4_, 14.7 mM KH_2_PO_4_, pH 6.9–7.1) or in phosphate limiting (PL) media (0.1% w/v (NH_4_)_2_SO_4_, 0.41 mM MgSO_4_, 0.2% w/v glucose, 0.1 M HEPES, pH 6.9–7.1 ± 5 mM KH_2_PO_4_) in shake flasks at 300 rpm, or on plates supplemented with 1.5% (w/v) agar (LBA). For the *phoBR *mutagenesis screen, *Serratia *39006 was grown on PGM agar plates (5 g l^-1 ^bacto peptone, 1% v/v glycerol and 1.5% w/v agar). Bacterial growth (OD_600_) was measured in a Unicam Heλios spectrophotometer at 600 nm. When required, media were supplemented with antibiotics at the following final concentrations; kanamycin 50 μg ml^-1 ^(Km), spectinomycin 50 μg ml^-1 ^(Sp), ampicillin 100 μg ml^-1 ^(Ap), and tetracycline 35 μg ml^-1 ^(Tc). The generalised transducing phage ϕOT8 was used for transduction of chromosomal mutations as described previously [25].

### DNA manipulations

All molecular biology techniques, unless stated otherwise, were performed by standard methods [50]. Oligonucleotide primers were obtained from Sigma Genosys and are listed in Table 1. DNA sequencing was performed at the DNA Sequencing Facility, Department of Biochemistry, University of Cambridge, analysed using GCG (Genetics Computer Group, University of Wisconsin) and compared with GenBank DNA or non-redundant protein sequence databases using BLAST [51].

**Table 1:** Oligonucleotide primers used in this study

Name	5'-3' sequence	Description	Restriction site
HS34	GCTGACTCATAAATATCTGACTG	*pigA*, primer extension oligo	
HS36	GCGAAAATAGCTCGGCTGATCTC	*smaI*, primer extension oligo	
HS60	GTCTATATCGGCATCTGTTCC	*carA*, primer extension oligo	
KML	CCAGTAAGTTTTCCAGTAGGTGG	F primer for Km^R ^gene of miniTn*5*Km1	
KMR	CCGAGCTTGGTACCCAGTC	R primer for Km^R ^gene of miniTn*5*Km1	
NW225	GACCACACGTCGACTAGTGCNNNNNNNNNACTG	Random primed PCR primer 1	
NW226	GACCACACGTCGACTAGTGCNNNNNNNNNATGAC	Random primed PCR primer 2	
NW227	GACCACACGTCGACTAGTGCNNNNNNNNNGTCTC	Random primed PCR primer 3	
NW244	CGTCTGCCAGGTGCTATTGGTTATG	*pstSCAB *region sequencing primer	
NW245	GGATAACGAAGTGAACAGCAAC	*pstSCAB *region sequencing primer	
NW246	GCATCCTGGCCGAGCATACAGAAG	*pstSCAB *region sequencing primer	
NW247	GCGACGCATGCGGATAAGCTCTG	*pstSCAB *region sequencing primer	
NW250	CATTACTGCGATGCACAATCAG	*phoU *sequencing primer	
NW251	GTGACGATTGATGAAGCTTGTG	*phoU *sequencing primer	
OTG124	ATCAGAGAATTCTACTAATTGGAGTCATTACCG	F primer for pTG27, *smaI *promoter construct	*Eco*RI
OTG125	ATCAGAAAGCTTAGTCTATCATTATAGCGTTCC	R primer for pTG27, *smaI *promoter construct	*Hin*dIII
PF42	GCATAAGCTTCCATCACTACTCC	R primer for pTA14, *rap *promoter construct	*Hin*dIII
PF43	GTAAGAATTCGCGATGTTCAGAAAC	F primer for pTA14, *rap *promoter construct	*Eco*RI
PF154	GATGAATTCAGGAGGACAGGGATGGCAAGACGTATTTTG	F primer for pTA74, PhoB expression construction	*Eco*RI
PF155	TCTAAGCTTCAGTAACGCGTCGAG	R primer for pTA74, PhoB expression construction	*Hin*dIII
PF180	TTTGAATTCGTTAGTTTGGGAGATTTTC	F primer for sequencing *phoR*	*Eco*RI
PF182	TTTAAGCTTGCTGCGGGACGC	R primer for sequencing *phoR*	*Hin*dIII
PHORL	GCGTTAGTTTGGGAGATTTTC	F *phoR *primer	
PHORR	CTCCCAAACTAACGCTGTC	R *phoR *primer	
PST1	CAGCGTCTGCCAGGTGC	*pstS *sequencing primer	
PST2	GTCCACGTTGCTGAG	*pstA *sequencing primer	
PST3	CCAGCTTTACCCAGAGCAACATG	*pstB *sequencing primer	
PST4	CAGAGTGTAGTTTGCAGG	*pstS *sequencing primer	
PST5	CGAGCAACAGCCAGTAG	*pstA *sequencing primer	
PSTSLN	CAACAGGATAAAGGTAGTGGAGG	*pstS *sequencing primer	
PSTSRN	CTGCACGGTCTTGGTCG	*pstS *sequencing primer	
T3	CGCGCAATTAACCCTCACTAAAG	pBluescript II KS+ sequencing primer	
T7	GCGCGTAATACGACTCACTATAG	pBluescript II KS+ sequencing primer	

### Sequencing of the *pstSCAB-phoU *operon

Preliminary sequence analysis indicated the mini-Tn*5*Sm/Sp insertions in strains ROP2 and KHC5 were in *pstS *and *pstA *respectively [29]. To determine the full sequence of *pstS *and its surrounding genes, a *Serratia *39006 *Pst*I sub-genomic library was created in pBluescript II KS+. One clone containing *pstS *was analysed further and was named pPST1. The *pst *region was sequenced via a 'primer walking' technique using primers PST1, PST2, PST3, PST4, PST5, PSTSLN, PSTSRN. To complete the *pstSCAB-phoU *operon, a 2.1 kbp region of *pstSCA *was PCR amplified with the primers NW244 and NW245, and then sequenced using primers NW244, NW245, NW246 and NW247. Random primed PCR was used to extend the *phoU *sequence obtained from primer walking of pPST1, as described previously [48]. Gene specific primer NW250 was used in two separate random primed PCR reactions, one with PF106, PF107, PF108 [48], and a second with NW225, NW226, NW227. The products generated were then amplified with the nested primer PF109 or NW251, respectively and the resulting PCR products sequenced with primer NW251.

### Transposon mutagenesis screen for *phoBR *mutants

To isolate *phoBR *mutants, *Serratia *39006 strain LacA was subjected to a random transposon mutagenesis by conjugation with *E. coli *S17–1 λ*pir *harbouring plasmid pUTmini-Tn*5*Km1 as described previously [25]. Ten thousand mutants were picked onto glucose minimal medium plates and replica-plated onto PGM agar Colonies that did not exhibit a hyper-pigmented phenotype were selected, based on the rationale that if hyper-pigmentation was not induced in response to P_i _limitation, it might be due to an insertion in *phoBR *(strains BR1 and BR9 were isolated using this screen). The *pstS*::miniTn5Sm/Sp was transduced into non-P_i _responsive mutants, and non-hyperpigmented mutants were then selected (strains RBR1 and RBR9 were selected following this screen). This suggested that these uncharacterised insertions had disrupted a regulatory element(s) common to *pstS *mutants and P_i _limitation effects. The possibility that *phoBR *had been disrupted was investigated further by measuring alkaline phosphatase activity, encoded by *phoA*, which is a well conserved member of enteric Pho regulons [1]. Mutants RBR1 and RBR9 did not produce elevated levels of alkaline phosphatase as observed in the *pstS *mutant (data not shown). Sequence analysis, described below, confirmed that the insertions in BR1 and BR9 were within *phoR *and *phoB *respectively.

### Sequencing of the *phoBR *operon

To determine the site of the transposon insertion in strain BR1, chromosomal DNA was digested with *Eco*RV and ligated into pBluescript II KS+. The ligation was used as template in a single-primer-site PCR using primers KML and KMR that anneal to the 5' and 3' ends of mini-Tn*5*Km1 respectively in combination with primers T3 and T7. Sequencing of the resultant PCR products revealed that BR1 contained an insertion within a gene similar to *phoR *from *E. coli*. A further PCR using chromosomal DNA from the BR9 mutant with primers PHORL and PHORR (homologous to *phoR *5' and 3' ends) and primers KML and KMR demonstrated that BR9 contained an insertion within a gene with similarity to *phoB *from *E. coli*. To further confirm the *phoBR *sequence, PCR products of *phoB *and *phoR *were generated with primer pairs PF154/PF155 and PF180/PF182 respectively and sequenced on both strands from independent products.

### Construction of a plasmid (pTA74) that expresses native PhoB

A construct that enabled expression of native, untagged PhoB was created as outlined below. The *phoB *gene was amplified by PCR, using primers PF154 and PF155, which contain *Eco*RI and *Hin*dIII restriction sites, respectively. Additionally, primer PF154 contains a consensus ribosome-binding site (RBS, AGGAGGA). The PCR fragment of *phoB *was cloned into pQE-80L, previously digested with the enzymes *Eco*RI and *Hin*dIII. The resulting plasmid, pTA74, was confirmed by DNA sequencing. Expression of plasmid pTA74 in *E. coli *was induced with 1 mM IPTG.

### Construction of promoter::*lacZ *fusions and assay conditions

Plasmid pTA15 was constructed as described previously [48]. The *rap *and *smaI *promoter regions were cloned into the promoterless *lacZ *plasmid pRW50 [49] to give the plasmid constructs pTA14 and pTG27, respectively. Plasmid pTG27 was constructed by cloning an *Eco*RI/*Hin*dIII digested PCR product (generated using forward primer OTG124 and reverse primers OTG125) into *Eco*RI/*Hin*dIII digested pRW50. Plasmid pTA14 was constructed by cloning an *Eco*RI/*Hin*dIII digested PCR product (generated using forward primer PF43 and reverse primer PF42) into *Eco*RI/*Hin*dIII digested pRW50. All constructs were confirmed by DNA sequencing.

Promoter activity assays were performed in *E. coli *DH5α cells as described in [48]. Briefly, DH5α cells were transformed with the promoter::*lacZ *construct (pTA14, pTA15 or pTG27) and either pTA74 (encoding native PhoB) or the empty vector control, pQE-80L. The resulting strains were grown in LB containing Ap, Tc and 1 mM isopropyl-β-D-thiogalactopyranoside (IPTG). At late exponential phase, 1 ml samples were assayed for β-galactosidase activity.

### Prodigiosin, carbapenem, AHL, β-galactosidase, β-glucuronidase and alkaline phosphatase assays

The assays for Pig and Car were performed as described previously [29]. Pig production was plotted as (A_534 _ml^-1 ^OD_600_^-1^). Detection of AHLs was performed using the *Serratia *LIS bioassay described in [25]. β-Galactosidase activity was determined as described previously [28] and was represented as (ΔA_420 _min^-1 ^ml^-1 ^OD_600_^-1^). β-Glucuronidase activity was determined as for β-galactosidase activity except that reactions were performed in GUS buffer (50 mM NaPO_4_, 1 mM EDTA, 5 mM DTT, pH 7.0), using the substrate *p*-nitrophenyl β-glucuronide (PNPG; 10 mM), and measured at A_405_. β-Glucuronidase activity was represented as (ΔA_405 _min^-1 ^ml^-1 ^OD_600_^-1^). Alkaline phosphatase activity was assayed as described previously [52]. Results presented are the mean ± the standard deviation of three independent experiments, unless stated otherwise.

### Primer Extension and RNA studies

RNA was extracted from *Serratia *39006 and primer extension analysis for the *pigA *and *smaI *transcripts was performed as described previously [28,29]. All primer extension reactions were performed with 25 μg of total RNA and 0.2 pmol of the appropriate ^32^P-labelled primer. Oligonucleotide primers HS34 and HS36 were used in primer extension reactions for *pigA *and *smaI *respectively.

## Authors' contributions

TG drafted the manuscript, participated in design of the study and performed all experiments that are not credited to the additional authors, listed below. PF generated multiple strains (PCF# strains) and plasmids used in the study, participated in sequencing *phoBR*, participated in design of the study and critically reviewed the manuscript. LE isolated strains BR1 and BR9, performed primer extension analysis, participated in sequencing *phoBR *and *pstSCAB-phoU*, and participated in design of the study. NW generated strain NW201 and NW202, measured *pstC*::*uidA *expression and participated in sequencing of *pstSCAB-phoU*. GS conceived of the study and participated in the design and coordination of the study.

## Supplementary Material

Additional file 1 **Bacterial strains, phages and plasmids used in this study**. A list of strains, phage and plasmids used in this study. Click here for file
