# Supplementary material for: The PhoBR two-component system regulates antibiotic biosynthesis in Serratia in response to phosphate
Source: BMC Microbiol. 2009 May 28;9:112. doi: 10.1186/1471-2180-9-112 (PMC2695467; doi:10.1186/1471-2180-9-112)
Supplement: Additional file 1 — Bacterial strains, phages and plasmids used in this study. A list of strains, phage and plasmids used in this study. [file 1471-2180-9-112-S1.doc]

**Additional file 1.** Bacterial strains, phages and plasmids used in this study.

| Strain/plasmid | Genotype/phenotype | Reference |
| --- | --- | --- |
| *Escherichia coli* |  |  |
| BW20767 | *RP4-Tc-::Mu-1, kan::Tn7* integrant*, leu63::IS10, recA1, zbf-5, creB510, hsdR17, endA1, thi, uidA (∆MluI)::pir^+^* | [44] |
| CC118 *λpir* | *araD, ∆(ara, leu), ∆lacZ74, phoA20, galK, thi-1, rspE, rpoB, argE, recA1, λpir* | [45] |
| DH5α | F^-^, Φ80*∆dlacZM15, ∆(lacZYA-argF)U169, endA1, recA1, hsdR17 (rκ^-^mκ^+^), deoR, thi-1, supE44, λ^-^, gyrA96, relA1* | Gibco/BRL |
| ESS | Β-lactam super sensitive indicator strain | [46] |
| SM10 *λpir* | *thi-1, thr, leu, tonA, lacY, supE, recA::RP4-2-Tc::Mu, λpir*, Km^R^ | [47] |
| S17-1 λpir | *recA, pro, hsdR, recA::RP4-2-Tc::Mu, λpir*, Tmp^R^, Sp^R^, Sm^R^ | [47] |
|  |  |  |
| *Serratia* |  |  |
| ATCC 39006 | Wild-type (Car^+^, Pig^+^) | [23] |
| LacA (parental strain) | Lac^-^ derivative of ATCC 39006, made by EMS mutagenesis | [25] |
|  |  |  |
| Mutants derived from LacA |  |  |
| BR1 | *phoR*::mini-Tn*5*Km, Km^R^ | This study |
| BR9 | *phoB*::mini-Tn*5*Km, Km^R^ | This study |
| HSPIG17 | *pigQ*::mini-Tn*5lacZ1*, Km^R^ | [28] |
| HSPIG17::R2 | *pstS::*mini-Tn*5*Sm/Sp, *pigQ*::mini-Tn*5lacZ1*, Sm^R^, Km^R^ | This study |
| HSPIG23 | *pigR*::mini-Tn*5lacZ1*, Km^R^ | [28] |
| HSPIG23::R2 | *pstS::*mini-Tn*5*Sm/Sp, *pigR*::mini-Tn*5lacZ1*, Sm^R^, Km^R^ | This study |
| HSPIG26 | *pigS*::mini-Tn*5lacZ1*, Km^R^ | [28] |
| HSPIG26::R2 | *pstS::*mini-Tn*5*Sm/Sp, *pigS*::mini-Tn*5lacZ1*, Sm^R^, Km^R^ | This study |
| HSPIG36 | *pigT*::mini-Tn*5lacZ1*, Km^R^ | [48] |
| HSPIG36::R2 | *pstS::*mini-Tn*5*Sm/Sp, *pigT*::mini-Tn*5lacZ1*, Sm^R^, Km^R^ | This study |
| HSPIG46 | *pigV*::mini-Tn*5lacZ1*, Km^R^ | [28] |
| HSPIG46::R2 | *pstS::*mini-Tn*5*Sm/Sp, *pigV*::mini-Tn*5lacZ1*, Sm^R^, Km^R^ | This study |
| HSPIG62 | *pigW*::mini-Tn*5lacZ1*, Km^R^ | [28] |
| HSPIG62::R2 | *pstS::*mini-Tn*5*Sm/Sp, *pigW*::mini-Tn*5lacZ1*, Sm^R^, Km^R^ | This study |
|  |  |  |
|  |  |  |
|  |  |  |
| Strain/plasmid | Genotype/phenotype | Reference |
|  |  |  |
| HSPIG67 | *pigP*::mini-Tn*5lacZ1*, Km^R^ | [28] |
| HSPIG67::R2 | *pstS::*mini-Tn*5*Sm/Sp, *pigP*::mini-Tn*5lacZ1*, Sm^R^, Km^R^ | This study |
| HSPIGROP4::R2 | *pstS::*mini-Tn*5*Sm/Sp, *pigX*::mini-Tn*5lacZ1*, Sm^R^, Km^R^ | This study |
| ISRAPL | *smaI*::mini-Tn*5*Sm/Sp, *rap*::mini-Tn*5lacZ1*, Sp^R^, Km^R^ | [28] |
| KHC5 | *pstA*::mini-Tn*5*Sm/Sp, Sp^R^ | [29] |
| LC13 | *smaI*::mini-Tn*5lacZ1*, Km^R^ | [25] |
| LIS | *smaI*::mini-Tn*5*Sm/Sp, Sp^R^ | [25] |
| MCA54 | *carA*::mini-Tn*5lacZ1*, Km^R^ | [25] |
| MCP2L | *pigA*::mini-Tn*5lacZ1*, Km^R^ | [29] |
| MCR14 | *carR*::mini-Tn*5lacZ1*, Km^R^ | [29] |
| NW60 | *pigA*::*lacZ*Cm, Cm^R^ | (Williamson *et al*., unpubl.) |
| NW201 | *pstC*::Tn-*uidA*Cm, Cm^R^ | This study |
| NW202 | *phoB*::mini-Tn*5*Km, *pstC*::Tn-*uidA*Cm, Km^R^ , Cm^R^ | This study |
| PCF45 | *pstS::*mini-Tn*5*Sm/Sp, *rap*::mini-Tn*5lacZ1*, Km^R^, Sp^R^ | This study |
| PCF58A9* | *pstC*::Tn-*uidA*Cm, Cm^R^ | This study |
| PCF59 | *pstS::*mini-Tn*5*Sm/Sp, *phoB*::mini-Tn*5*Km, Sp^R^, Km^R^ | This study |
| PCF60 | *pstS::*mini-Tn*5*Sm/Sp, *phoR*::mini-Tn*5*Km, Sp^R^, Km^R^ | This study |
| PCF74 | *pigA*::*lacZ*Cm, *phoB*::mini-Tn*5*Km, Cm^R^, Km^R^ | This study |
| PCF75 | *pigA*::*lacZ*Cm, *phoR*::mini-Tn*5*Km, Cm^R^, Km^R^ | This study |
| PCF76 | *pigA*::*lacZ*Cm, *pstS*::mini-Tn*5*Sm/Sp, Cm^R^, Sp^R^ | This study |
| PCF77 | *pigA*::*lacZ*Cm, *phoB*::mini-Tn*5*Km, *pstS*::mini-Tn*5*Sm/Sp, Cm^R^, Km^R^, Sp^R^ | This study |
| PCF78 | *pigA*::*lacZ*Cm, *phoR*::mini-Tn*5*Km, *pstS*::mini-Tn*5*Sm/Sp, Cm^R^, Km^R^, Sp^R^ | This study |
| RAPL | *rap*::mini-Tn*5lacZ1*, Km^R^ | [28] |
| RAPS | *rap*::mini-Tn*5*Sm/Sp, Sp^R^ | [28] |
| RBR1 | *pstS::*mini-Tn*5*Sm/Sp, *phoR*::mini-Tn*5*Km, Sp^R^, Km^R^ | This study |
| RBR9 | *pstS::*mini-Tn*5*Sm/Sp, *phoB*::mini-Tn*5*Km, Sp^R^, Km^R^ | This study |
| ROP2 | *pstS*::mini-Tn*5*Sm/Sp, Sp^R^ | [29] |
| ROP4 | *pigX*::mini-Tn*5lacZ1*, Km^R^ | [28] |
| TG39 | *pigZ*::mini-Tn*5lacZ1*, Km^R^ | [36] |
| TG71 | *pstC*::Tn-*uidA*Cm, *smaI*::mini-Tn*5*Sm/Sp, *rap*::mini-Tn*5lacZ1*, Cm^R^, Sp^R^, Km^R^ | This study |
|  |  |  |
|  |  |  |
|  |  |  |
|  |  |  |
| Strain/plasmid | Genotype/phenotype | Reference |
|  |  |  |
| Phage |  |  |
| ΦOT8 | *Serratia* generalized transducing phage | (Crow *et al.* unpubl.) |
|  |  |  |
| Plasmids |  |  |
| pBluescript II KS+ | Cloning vector, ColE1 replicon, Ap^R^ | Stratagene |
| pDS1028*uidA* | Delivery plasmid for Tn-*uidA*Cm, derivative of pDS1028, Tc^R^, Cm^R^ | (Williamson *et al*., unpubl.) |
| pNRW112 | Delivery plasmid for Tn-DS1028*lacZ*Cm, derivative of pDS1028, Tc^R^, Cm^R^ | (Williamson *et al*., unpubl.) |
| pPST1 | *Serratia* 39006 *Pst*I subgenimic clone encoding *pstS*, pBluescript II KS+ derivative, Ap^R^ | This study |
| pRW50 | Promoterless *lacZ* fusion plasmid, RK2 replicon, Tc^R^ | [49] |
| pTA14 | *rap* promoter *lacZ* fusion (-301 to +6), Tc^R^ | This study |
| pTA15 | *pigA* promoter *lacZ* fusion (-367 to +49), Tc^R^ | [48] |
| pTA74 | PhoB expression vector, pQE-80L derivative, Ap^R^ | This study |
| pTG27 | *smaI* promoter *lacZ* fusion (-238 to +10), Tc^R^ | This study |
| pUTmini-Tn*5*Km1 | Delivery plasmid for mini-Tn*5*Km1, Ap^R^, Km^R^ | [47] |
| pQE-80 L | Cloning vector, Ap^R^ | QIAGEN |

*Strain PCF58A9 was created as part of a separate study (P. Fineran, unpublished results) and the accurate genotype is *pstC*::Tn-*uidA*Cm, *pigV*::mini-Tn*5lacZ1*, *pigN*::*blaM*, Cm^R^, Km^R^. However, for this study, PCF58A9 was used to generate a phage lysate (using ΦOT8) in order to transduce only the *pstC*::Tn-*uidA*Cm mutation into strains LacA, BR9 or ISRAPL in order to generate strains NW201, NW202 and TG71.
